# Exploring demands of hemodialysis patients in Taiwan: A two-step cluster analysis

**DOI:** 10.1371/journal.pone.0228259

**Published:** 2020-02-07

**Authors:** I-Chen Yu, Ji-Tseng Fang, Yun-Fang Tsai

**Affiliations:** 1 Department of Nursing, Chang Gung University of Science and Technology, Taoyuan, Taiwan; 2 Department of Nephrology, Chang Gung Memorial Hospital, Taoyuan, Taiwan; 3 College of Medicine, Chang Gung University, Taoyuan, Taiwan; 4 School of Nursing, College of Medicine, Chang Gung University, Taoyuan, Taiwan; 5 Department of Psychiatry, Chang Gung Memorial Hospital, College of Medicine, Chang Gung University, Keelung, Taiwan; East Carolina University Brody School of Medicine, UNITED STATES

## Abstract

**Aims and objectives:**

To classify hemodialysis patients into subgroups via cluster analysis according to the Somatic Symptoms Disturbance Index, Taiwanese Depression Scale, and Herth Hope Index scores. Patient demands in each cluster were also examined.

**Background:**

Overall patient demands among hemodialysis patients have been demonstrated in numerous reports; however, variables among subgroups have not been explored.

**Methods:**

Data were analyzed from a cross-sectional survey of 114 hemodialysis patients recruited from dialysis centers in Northern Taiwan. Hope, depression, and symptom disturbance were used as parameters for clustering because they have been shown to be important factors affecting patient demands. A two-step cluster analysis was performed to classify participants into clusters. Patient demands in each cluster were analyzed.

**Results:**

Among the 114 participants, there was a negative correlation between hope and depression as well as between hope and symptom disturbance; there was a positive correlation between depression and symptom disturbance. Two clusters were identified: Cluster 1 (n = 49) included patients with moderate levels of hope and symptom disturbance, and high levels of depression; and Cluster 2 (n = 65) included patients with low levels of depression and symptom disturbance and high levels of hope. Demographic profiles differed between the two clusters. Regarding patient demands, medical demand showed the highest average score; whereas, occupational demand exhibited the lowest average score. Psychological and occupational demands differed significantly between the two clusters. The two clusters were defined as subgroups: Cluster 1 was labeled “resting”; Cluster 2 was labeled “active”.

**Conclusions:**

Cluster analysis may further classify hemodialysis patients into distinct subgroups base on their specific patient demands. A better understanding of patient demands may help health professionals to provide a holistic individualized treatment to improve patients’ outcomes.

## Introduction

End stage renal disease (ESRD) is a chronic condition which threatens patient health worldwide. Patients rely on either maintenance dialysis or renal transplant; most choose the former. In 2015, a total of 83,808 patients in Taiwan received hemodialysis treatment [1], which is a lengthy process requiring two to three 4-hour sessions per week for the duration of the patient’s life. Consequently, hemodialysis results in physical, psychological, and social stresses [2], and a reduction in quality of life (QOL) [3–5]. Common physical discomforts include tiredness, dry mouth, lack of vitality, and muscle weakness [6]. One study demonstrated the severity of discomforts, such as fatigue, itchiness, thirst, joint pain, and sleep disruption, were positively correlated with anxiety and depression [7].

Depression is the most common psychological issue for dialysis patients [8]. Depression affects symptom disturbance and overall health [6]; one study reported that in approximately 60% of hemodialysis patients, depression was the result of their disease [9]. Depression may induce immune deficiency, loss of appetite, and malnutrition, rendering patients unable to cope with their prescribed therapy; depression can also provoke suicide, thus indirectly raising the mortality rate [10]. Gender, age, and comorbidity have been recognized as critical factors leading to depression [11]; lack of social support and a lengthy treatment regimen may also result in depression [12,13].

The subjective perception of hope can have a positive influence on health outcomes for patients with a chronic disease [14]. For hemodialysis patients, hope is influenced by a patient’s perception of the severity and prognosis of their disease, as well as the subjective perception of their health condition; higher levels of hope have been shown to be positively correlated with better patient outcomes [15,16]. Billington et al. surveyed the mental status of hemodialysis patients and demonstrated that ‘hope’ was a predictor of depression, anxiety, symptom burden and QOL; the higher the level of hope, the lower the level of depression and symptom disturbance, accompanied with better mental health and QOL [17]. When hope was assessed in 50 older adult hemodialysis patients [18] using the Herth Hope Index (HHI), scores were lower than for patients with chronic obstructive pulmonary disease [19] and heart failure [20]; in addition, religious beliefs were positively correlated with the degree of hope [18]. Another study demonstrated scores on the HHI were positively correlated with sprituality in patients undergoing hemodialysis, suggesting both constructs, hope and spirituality, might help patients cope with chronic kidney disease [21]. Hope has also been shown to be positively correlated with physical functioning and negatively correlated with depression in patients with oral cancer [22]. Consequently, people with high levels of hope and a low levels of depression experience enhanced physical functioning [23–25]. These studies highlight the benefits of hope for patients with a chronic disease.

Understanding the demands of hemodialysis patients could help these patients cope with this chronic disease and help nurses provide appropriate support to increase hope. Studies have examined the demands of family members of dialysis patients [26] and the general needs of dialysis patients [27]. However, demands of patients may vary with age, severity of physical symptoms, and psychological state. Demands can also be categorized according to patient characteristics. Therefore, the objectives of this study were to classify hemodialysis patients into subgroups using cluster analysis according to three important patient characteristics: symptom disturbance, depression, and hope; and to examine patient demands within these subgroups

## Materials and methods

### Participants

This cross-sectional study was conducted in a dialysis clinic in Northern Taiwan. Patients were selected by convenience sampling. We recruited patients who satisfied the following inclusion criteria: (a) having been receiving hemodialysis treatment for at least three months; (b) age between 20–80 years old; (c) being alert and oriented; and (d) a good comprehension of Taiwanese or Mandarin. Patients with cognitive disorders or in a critical condition were excluded from the study. The study was performed and reported in compliance with the STROBE guidelines [28].

### Procedures

This study was approved by the institutional review board of Chang Gung Memorial Hospital (IRB No.94-630). The clinical physicians and nurses in the dialysis clinic were asked to refer patients who met the inclusion criteria. The first author, who had a doctoral degree in nursing and had been working with this specific patient group for over 10 years, explained the purpose and procedures of the study. The description included the time that would be required to complete the survey (about 20 to 30 minutes), that the survey would be conducted following dialysis, and that confidentiality of collected data would be maintained as required by the regulations of the IRB. The researcher explained that the data would be stored in a locked cabinet designated to be used exclusively for the survey and would be kept for 3 years after completion of the study. Data files were de-linked and encoded to assure confidentiality. Patients were also assured they could withdraw from the study at any time and for any reason without their care being compromised.

Patients who expressed a willingness to participate and provided signed informed consent were provided with the survey questionnaires. This survey was conducted on paper anonymously. Participants were asked to complete instruments regarding demographic and medical characteristics, self-report scales; no other personal information was collected. Transportation was subsidized by providing participants the equivalent of 15 USD.

### Instruments

Data were collected using instruments for demographic and medical characteristics, the somatic symptoms disturbance index (SSDI), the Taiwanese depression scale (TDS), the Herth hope index (HHI), and the hemodialysis patients demand scale (HDS). These instruments are described in the following sections.

#### Demographic and medical characteristics

A questionnaire was disturbed to all participants regarding demographic and medical information. Demographic data included age, gender, educational level, occupation, marital status, and religion. Medical information included duration of dialysis, and clinical data regarding blood urea nitrogen (BUN), creatinine, electrolytes, hemoglobin, and albumin levels.

#### Somatic Symptoms Disturbance Index (SSDI)

We used a modified version of the SSDI scale developed by Weisbord et al. [29] to assess the severity of physical symptoms; patients are asked to report the appearance of symptoms and the severity of each symptom during the previous week. The modified SSDI scale consists of 23 items, which describe physical symptoms. Severity is rated on a 4-point Likert scale (0 = absent; 1 = mild; 2 = moderate; 3 = severe); scores range from 0 to 69, with higher scores indicating greater symptom disturbance. In this study, the Cronbach’s α was 0.86 and the content validity index (CVI) was 0.8.

#### Taiwanese Depression Scale (TDS)

The Taiwanese Depression Scale (TDS) was developed for psychiatric patients with depression and anxiety [30]. The 18-item TDS is a self-report instrument used widely as a screening tool for depression in Taiwan; reliability and validity have been well-established, with a Cronbach’s α of 0.90. Frequency over the previous week is reported for symptoms of depression, including emotional and behavioral symptoms, using a 4-point Likert scale: 0 = never or rarely (less than one day), 1 = occasionally (1 to 2 days), 2 = often (3 to 4 days), and 3 = frequently (5 to 7 days). Total scores range from 0 to 72 and depression is then divided into four levels according to the total score obtained: < 8, a stable emotional state; 9–14, the emergence of depression; 15–18, heightened depression; 19–28, a critical level of depression at which immediate medical or psychological help is advised; and >29, a serious level of depression that is in urgent need of specialized medical care. Using a cut-off score of 19, the sensitivity was 0.89 and the specificity was 0.92. The Cronbach’s α of this study was 0.90 and the CVI was above 0.85.

#### Herth Hope Index (HHI)

The HHI is a modified and an abbreviated version of the Herth hope scale (HHS) developed by Herth [14]. The HHI consists of 12 items; each item is rated on a 4-point Likert scale. The HHI contains positively and negatively worded items. The positively worded items are scored from 4 (*strongly agree*) to 1 (*strongly disagree*); and the negatively ones are scored in reverse. The Chinese version of the HHI was used in this study [31]. The total score ranges from 12 to 48, with higher scores indicating greater levels of hope. The Cronbach’s α for this study was 0.70 and the CVI was above 0.8.

#### Hemodialysis Patients Demand Scale (HDS)

The 27-item HDS was developed and modified by Huang et al. to measure demands of hemodialysis patients in Taiwan [32]; each item is scored on a 4-point Likert scale ranging from 0 (no demand) to 3 (strong demand). The total score ranges from 0 to 81, with higher scores indicating greater demands. This scale has been demonstrated to have excellent reliability and validity, with a Cronbach’s α for internal consistency of 0.91. Factor analysis testing was employed for validity testing, and seven factors were extracted from the scale describing patient demands: physiological, psychological, medical, occupational, informational, social, and financial. The seven factors explained 65.05% of the variance of the scale. The Cronbach’s α for this study was 0.92 and the CVI was above 0.85.

### Sample size calculation

Sample size in investigative research can be calculated as 5 times of the number of items in the questionnaire. To perform factor analysis, the minimum sample number required is 100 [33]. The lowest number of items in these instruments was 18; therefore, the minimum sample size required was 90. A total of 114 patients were registered in this study, indicating adequate sample size.

### Data analysis

The raw data from the questionnaires were collected, processed, encoded, and input into a computer for analysis with SPSS/PC for Window 23.0 statistical software. All the data were analyzed by applying descriptive statistics and inference statistics including a *t* test and cluster analysis, which was performed to classify patients. Cluster analysis was used to divide data into clusters with elevated within-cluster homogeneity and between-cluster heterogeneity [34]. A 2-step clustering was applied in order to cluster patients in this study. The patients were partitioned according to their SSDI, TDS, and HHI scores. The between-cluster differences regarding patient demands were subsequently compared. Spearman’s Correlation Coefficient was used to verify the existence and magnitude of correlation among SSDI, TDS, and HHI. In the present study, the magnitude of correlations was defined as: weak (0.1 to 0.39), moderate (0.4 to 0.69), and strong (0.7 to 0.99) [35].

## Results

### Demographic characteristics and medical information

Of the patients who met the inclusion criteria, 116 patients agreed to participate in this study; 2 participants did not complete the questionnaires. Therefore, date were analyzed for a total of 114 participants. Among the participants, 57.9% were male with an average age of 52.43 years, and a mean duration of dialysis treatment of 6.51 years. A total of 48.2% of participants received at least 12 years of education; a quarter of participants reported having no religious beliefs. Thirty percent of the patients were still employed. Abnormal blood biochemical parameters, including BUN, Cr, K, P, and HGB, were detected in the vast majority of the participants, as listed in Table 1.

**Table 1:** Demographic and medical characteristics of total participants (N = 114), Cluster 1 (n = 49), Cluster 2 (n = 65) and differences between the two clusters.

	Total Participants		Cluster 1		Cluster 2			
Characteristic	N (%)	M(SD)	n (%)	M(SD)	n (%)	M(SD)	t/χ^2^	p
Demographic								
Gender								
Male	66 (57.9)		16 (32.7)		50 (76.9)			
Female	48 (42.1)		33 (67.3)		15 (23.1)			
Age (years)		52.43(11.89)		55.34(11.52)		50.23(11.77)	2.32	0.02*
Education								
< 12 years	59 (51.8)		31 (63.3)		28 (43.1)		4.56	0.03*
**≥** 12-years	55 (48.2)		18 (36.7)		37 (56.9)			
Religion							0.41	0.53
Yes	85 (74.6)		38 (77.6)		47 (72.3)			
No	29 (25.4)		11 (22.4)		18 (27.7)			
Employed							7.22	0.007**
Yes	45 (39.5)		12 (24.5)		33 (50.8)			
No	69 (60.5)		37 (75.5)		32 (49.2)			
Medical								
Dialysis duration (years)		6.51(6.35)		7.19(6.83)		6.00(5.96)	0.96	0.32
Clinical data								
BUN (mg/dL) range = 6–21		75.01(17.91)		76.07(19.44)		74.28(16.94)	0.49	0.63
Cr (mg/dL); range = 0.64–1.27		12.17(6.56)		10.79(2.32)		13.11(8.18)	-1.74	0.84
Na (mEq/L); range = 134–138		139.08(3.11)		139.34(3.14)		138.90(2.90)	0.65	0.52
K (mEq/L); range = 3.6–5.0		5.10(0.74)		5.29(0.68)		4.97(0.76)	2.12	0.37
P (mEq/L); range = 2.4–4.7		4.90(1.60)		4.64(1.55)		5.07(1.61)	-1.24	0.22
Platelets/μl, range = 150–400		198.49(53.78)		193.62(63.10)		201.80(46.71)	-0.69	0.49
ALB (g/dL), range = 3.5–5.0		4.01(0.43)		3.87(0.43)		4.09(0.37)	-2.77	0.007**
HGB (g/dL) range = 12–16		10.20(1.40)		9.94(1.25)		10.37(1.48)	-1.51	0.14

M = mean; SD, standard deviation; BUN, blood urea nitrogen; Cr, creatinine; Na, sodium; K, potassium; P, phosphorous; PLATE, platelets; ALB, albumin; HGB, hemoglobin

### Correlation between hope, depression, and symptom disturbance among hemodialysis patients

The mean scores for hope, depression, and symptom disturbance for all participants were 34.08 (SD = 6.68), 8.56 (SD = 7.94), and 7.57 (SD = 5.99), respectively. Correlation analysis demonstrated a weak, but negative correlation between hope and depression (r = -.327, p< .001) as well as between hope and symptom disturbance (r = -.226, p< .05); whereas, a moderate positive correlation was shown between depression and symptom disturbance, as illustrated in Table 2.

**Table 2:** Correlations of hope, depression, and symptom disturbance among participants.

	Score		
Score	Hope	Depression	Symptom disturbance
Hope	1	-.327^**^	-.226^*^
Depression	--	1	.670^**^
Symptom disturbance	--	--	1

* p < .05

** p < .01

### Characteristics of the clusters

A 2-step cluster analysis was performed to classify participants according to scores on the SSDI, TDS, and HHI; two clusters were identified. Table 3 shows the scores for Cluster 1 (n = 49) and Cluster 2 (n = 65) and significant differences in scores between the two clusters. The mean scores for depression and symptom disturbance were significantly higher for Cluster 1 than Cluster 2 (p < .01) whereas the mean score for hope was significantly higher in Cluster 2 than Cluster 1 (p < .01). In addition, 24.5% of participants in Cluster 1 had a depression score higher than the cutoff point of 19 [29]; in contrast, all depression scores for participants in Cluster 2 were below the cutoff point of 19. The significant differences between these two clusters in all three characteristics confirm the effectiveness of the clustering approach.

**Table 3:** Scores for hope, depression and symptom disturbance by cluster analysis.

	Cluster 1 (n = 49)		Cluster 2 (n = 65)		
Score	Mean	SD	Mean	SD	*P*-value
Hope	31.61	4.60	35.02	5.26	< .01
Depression	14.85	7.78	3.81	3.55	< .01
Symptom disturbance	13.20	4.36	3.32	2.53	< .01

SD = standard deviation.

### Participant scores for hemodialysis patient demands

The HDS measured seven types of demands for hemodialysis patients: psychological, physiological, medical, financial, social, occupational, and information. The average total score on the HDS for our participants was 35.11 (SD = 17.24). The mean and average scores for the demands and their rank order are listed in Table 4. While medical demand showed the highest average score (1.72), occupational demand exhibited the lowest average score (0.83).

**Table 4:** Scores and rank for patient demands among all participants (N = 114).

	Score			
Patient demand	Mean	SD	Average score	Rank order
Psychological	8.78	5.10	1.46	3
Physiological	8.79	4.96	1.47	2
Medical	5.18	2.55	1.72	1
Financial	2.94	2.34	0.98	6
Social	4.76	4.18	1.19	5
Occupational	2.50	2.02	0.83	7
Information	2.87	1.66	1.44	4

SD, standard deviation

### Associations between each cluster, demographic profile and patient demands

There were significant differences in demographic characteristics between the two clusters (Table 1). The mean age of the participants in Cluster 1 was greater than the mean age of Cluster 2 (t = 2.32, p < .05); 57% of participants in Cluster 2 had ≥12 years of education compared with 37% in Cluster 1 (p = 0.03). While 51% of patients in Cluster 2 were employed, most participants in Cluster 1 (76%) were unemployed (p = 0.007).

When patient demands for the two clusters were compared (Table 5) psychological and occupational demands were significantly different between clusters. Compared with Cluster 2, the mean score for psychological demands was significantly greater for participants in Cluster 1 (p < .05) and lower for occupational demands (p < .05). There were no significant differences between the two clusters for any other demands (S1 Fig). Based on the above findings, Cluster 1 was labeled the “resting subgroup” and Cluster 2 was labeled the “active subgroup”.

**Table 5:** Differences in mean scores between patient demands for Cluster 1 (n = 49) and Cluster 2 (n = 65).

	Cluster 1		Cluster 2		
Patient demand	Mean	SD	Mean	SD	T
Psychological	9.93	4.77	7.91	5.20	.03*
Physiological	8.94	4.82	8.69	5.09	.79
Medical	5.41	2.45	5.01	2.63	.41
Financial	3.25	2.25	2.72	2.41	.24
Social	4.88	3.29	4.68	4.77	.80
Occupational	1.34	1.26	3.36	2.06	<. 01**
Information	3.04	1.68	2.75	1.65	.36

SD = standard deviation

*, p < .05

**, p < .01

## Discussion

This study examined levels of hope, depression, and symptom disturbance and patient demands among patients receiving hemodialysis for end-stage renal disease. Unlike past reports, this study classified patients by categorizing them according to profiles of hope, depression, and symptom disturbance using cluster analysis, which resulted in two distinct clusters: Cluster 1 and Cluster 2. When the two clusters were compared, there were significant differences in not only demographics, but also for psychological and occupational patient demands between Cluster 1 and Cluster 2.

### Hope, depression, and symptom disturbance for hemodialysis patients

For all participants, there were significant correlations between hope, depression, and symptom disturbance; levels of hope were inversely correlated with depression and symptom disturbance. Specifically, depression increased with the severity of symptom disturbance with concomitant decreases in levels of hope. The mean score for “hope” for participants in our study (34.08) was moderately higher than the mean score (32.72) reported Lin et al. [31]. Ottaviani et al. [21] found a positive correlation between hope and religious beliefs in patients with chronic kidney disease undergoing hemodialysis; 97% of patients attributed their hope to their religious beliefs. However, although most participants in our study (75%) reported having religious beliefs, this variable did not differ between the two clusters. The higher mean score for hope in Cluster 2, suggests the presence of religious beliefs was not a variable for participants in our study.

Religious beliefs have also been shown to impact depression and QOL. Kao et al. [36] reported that for patients receiving peritoneal dialysis in Taiwan, absence of religious beliefs was associated with low levels of depressive symptoms and high scores for QOL, while the magnitude of religious activity was negatively correlated with severity of depression. Our cluster analysis suggests religious beliefs may have little impact on variables of depression for hemodialysis patients with ESRD.

The mean score for depression was significantly lower for participants in Cluster 2 compared with Cluster 1. One explanation may be the significant difference in the demographic variable of employment and average monthly income between clusters, which was significantly higher for participants in Cluster 2 compared with Cluster 1. Vocational activity is important for patients since it may enhance their sense of personal value, increase social activities, and provide goals and meaning to life, thereby guiding them towards a positive psychological state. A survey of 861 hemodialysis patients in Taiwan showed that health-related quality of life (HRQOL) as well as mental health is positively correlated with higher monthly income and increased social activities [37]. Studies in the United States, Saudi Arabia, Japan and Greece also demonstrated that the severity of depression was negatively correlated with socioeconomic status in patients undergoing hemodialysis [38–41], indicating that correlation between depression and socioeconomic status could be a common phenomenon globally among this group of patients. The higher scores for depression in Cluster 1 might be explained by the significantly older age of participants compared to Cluster 2. Although age has been shown to play a role in emotional needs of hemodialysis patients [26], whether these needs are related to depression will require further studies.

### Hemodialysis patient demands

Regarding patient demands, all participants ranked medical demands highest of the seven items, followed by physiological and psychological demands. Medical demands encompass understanding future treatment plans, the type of information provided by medical personnel, and how to cooperate with medical personnel. Our findings demonstrating the high medical, physiological, and psychological patient demands are consistent with previous studies demonstrating medical demands among hemodialysis patients are closely related to physical and psychological conditions [27,32]. Although financial demands were ranked sixth, this should not be surprising for hemodialysis patients in Taiwan because most of the cost of hemodialysis care is paid by the National Medical Insurance program [1]. In addition, hemodialysis patients can apply for disability cards and receive a monthly cost of living subsidy of 3,000 to 6,000 NT dollars per month (the equivalent of 100 to 200 USD), depending on their financial status. Therefore, the social welfare policy may contribute to the observation of low financial demands.

Occupational demands ranked lowest for all participants in our study, and which is most likely due to the fact that 61% of participants (n = 69) were unemployed. The association between unemployment and occupational demands was further confirmed by the demographics of the two clusters. Occupational demands ranked lowest for participants in Cluster 1, which had significantly more unemployed participants (75%) compared with Cluster 2 (49%). In Taiwan, income for unemployed persons is provided by pensions, government subsidies, or family support. Thus, occupational demand would be low for this cluster. While the all participants ranked psychological demands third, psychological demands were significantly higher for Cluster 1, which might also be related to the greater scores for depression for participants in Cluster 1. As mentioned above, participants in Cluster 1 were older, which may also contribute to the increase in psychological demands [8].

We examined the demographics and patient demands of the two clusters in order to determine if there might be some commonalities that differentiated Cluster 1 from Cluster 2. Cluster 1 differed from Cluster 2 in that most participants who were unemployed, older, high psychological demands and low occupational demands. We labeled the Cluster 1 subgroup of participants as “resting” and the Cluster 2 subgroup as “active”. Previous studies on treatment of diseases or clinical care predominantly focused on specific patient groups. Cluster analysis provides a comprehensive model to classify patient demands according to various particular and important characteristics. In addition, this model has been recognized for its ability to provide clinical practitioners with specific parameters as the focus for implementing more effective intervention plans [42], in contrast to analyzing general, uncategorized parameters, which may not be as effective. Identification of these two clusters can help healthcare professionals better understand what variables play important roles for the improvement of patient outcomes.

This study had some limitations. First, our cross-sectional design limits our understanding of cause and effect, which will require further longitudinal studies to implement targeted strategies to improve outcomes. Second, our demographic questionnaire did not include questions regarding of different levels of education, income, or family support, which might have provided information regarding interactions between demographic variables and patient demands, which might have resulted in more homogeneous clusters.

## Conclusion

Our cluster analysis identified two groups of patients based on differences in scores for hope, depression and symptom disturbance. In addition, the two clusters differed significantly in scores for psychological and occupational demands. Understanding patient demands is fundamental to implementation of suitable and adequate care. Our findings could be used to design individualized treatment options for hemodialysis patients. Future studies should be conducted to examine what factors might differentiate these two clusters in order to design interventions that could increase hope, reduce depression, and improve treatment outcomes for hemodialysis patients. Although the scope of this study is limited to a national sample of patients in Taiwan, it provides a reference for other researchers investigating patients with end-stage renal disease.

## Supporting information

S1 Fig Patient demands for the two clusters of participants. (PDF)

## References

[1] National Health Insurance Administraion, Taiwan. (2016). http://www.nhi.gov.tw/Content_List.aspx?n=558D74136CD3253A&topn=CDA985A80C0DE710

[2] Abdel-Kader K, Unruh ML, Weisbord SD. (2009). Symptom burden, depression, and quality of life in chronic and end-stage kidney disease. Clinical Journal of The American Society of Nephrology: CJASN, 4(6), 1057–1064. 10.2215/CJN.00430109 19423570 PMC2689883

[3] Garcia RSA, Lucinda LMF, Ramos FA, Bueno GDS, de Oliveira GMR, Bonisson LSA, et al (2017). Factors associated with functional capacity in hemodialysis patients. *Artificial Organs*, 41(12), 1121–1126. 10.1111/aor.12938 28568475

[4] Liu X, Yang X, Yao L, Zhang Q, Sun D, Zhu X, et al (2017). Prevalence and related factors of depressive symptoms in hemodialysis patients in northern China. *BMC Psychiatry*, 17(1), 128 10.1186/s12888-017-1294-2 28381216 PMC5382415

[5] Shahgholian N, Tajdari S, Nasiri M. (2012). Reviewing and comparing self-concept in patients undergoing hemodialysis and peritoneal dialysis. *Iranian Journal of Nursing and Midwifery Research*, 17(2 Suppl 1), S85–S90. 23833607 PMC3696965

[6] Yu I-C, Huang J-Y, Tsai Y-F. (2012). Symptom cluster among hemodialysis patients in Taiwan. *Applied Nursing Research*, 25(3), 190–196. 10.1016/j.apnr.2010.11.002 21273045

[7] Alvarez-Ude F, Fernández-Reyes MJ, Vázquez A, Mon C, Sánchez R, Rebollo P. (2001). Physical symptoms and emotional disorders in patient on a periodic hemodialysis program. Nefrologia: Publicacion *Oficial De La Sociedad Espanola Nefrologia*, 21(2), 191–199. 11464653

[8] Kimmel PL, Peterson RA. (2005). Depression in end-stage renal disease patients treated with hemodialysis: tools, correlates, outcomes, and needs. *Seminars in Dialysis*, 18(2), 91–97. 10.1111/j.1525-139X.2005.18209.x 15771651

[9] Lu S-C, Chen S-I, Huang S-J, Su L-H. (2010). Analysis of sleep disturbance, depression and quality of life in maintenance hemodialysis patients. *Journal of Nursing and Healthcare Research*, 6(1), 33–43.

[10] Chen I-M, Lin P-H, Wu V-C, Wu C-S, Shan J-C, Chang S-S, et al (2018). Suicide deaths among patients with end-stage renal disease receiving dialysis: a population-based retrospective cohort study of 64,000 patients in Taiwan. *Journal of Affective Disorders*, 227, 7–10. 10.1016/j.jad.2017.10.020 29045916

[11] Chan L, Tummalapalli SL, Ferrandino R, Poojary P, Saha A, Chauhan K, et al (2017). The effect of depression in chronic hemodialysis patients on inpatient hospitalization outcomes. *Blood Purification*, 43(1–3), 226–234. 10.1159/000452750 28114133 PMC5823516

[12] Lilympaki I, Makri A, Vlantousi K, Koutelekos I, Babatsikou F, Polikandrioti M. (2016). Effect of perceived social support on the levels of anxiety and depression of hemodialysis patients. Materia Socio-Medica, 28(5), 361–365. 10.5455/msm.2016.28.361-365 27999485 PMC5149439

[13] Murillo-Zamora E, Macías-de la Torre A A, & Higareda-Almaraz MA. (2016). Depression prevalence among end stage renal disease patients in maintenance hemodialysis. *Revista Medica Del Instituto Mexicano Del Seguro Social*, 54(4), 429–433. 27197098

[14] Herth K. (1992). Abbreviated instrument to measure hope: development and psychometric evaluation. *Journal of Advanced Nursing*, 17(10), 1251–1259. 10.1111/j.1365-2648.1992.tb01843.x 1430629

[15] Cha J. (2017). Structural equation modeling of self-management in patients with hemodialysis. *Journal of Korean Academy of Nursing*, 47(1), 14–24. 10.4040/jkan.2017.47.1.14 28262651

[16] Rahimipour M., Shahgholian N., & Yazdani M. (2015). Effect of hope therapy on depression, anxiety, and stress among the patients undergoing hemodialysis. *Iranian Journal of Nursing and Midwifery Research*, 20(6), 694–699. 10.4103/1735-9066.170007 26793255 PMC4700689

[17] Billington E, Simpson J, Unwin J, Bray D, Giles D. (2008). Does hope predict adjustment to end-stage renal failure and consequent dialysis? *British Journal of Health Psychology*, 13(4), 683–699. 10.1348/135910707X248959 17958929

[18] Orlandi FDS, Pepino BG, Pavarini SCI, Dos Santos DA, de Mendiondo MSZ. (2012). The evaluation of the level of hope of elderly chronic kidney disease patients undergoing hemodialysis. *Revista Da Escola De Enfermagem Da U S P*, 46(4), 900–905. 10.1590/s0080-62342012000400017 23018400

[19] Alberto J, Joyner B. (2008). Hope, optimism, and self-care among Better Breathers Support Group members with chronic obstructive pulmonary disease. *Applied Nursing Research*, 21(4), 212–217. 10.1016/j.apnr.2006.12.005 18995163

[20] Rustøen T, Howie J, Eidsmo I, Moum T. (2005). Hope in patients hospitalized with heart failure. *American Journal of Critical Care*, 14(5), 417–425. 16120893

[21] Ottaviani AC, Souza ÉN, Drago NDC, de Mendiondo MSZ, Pavarini SCI, Orlandi FDS. (2014). Hope and spirituality among patients with chronic kidney disease undergoing hemodialysis: a correlational study. *Revista Latino-Americana De Enfermagem*, 22(2), 248–254. 10.1590/0104-1169.3323.2409 26107832 PMC4292610

[22] Hassanein KA, Musgrove BT, Bradbury E. (2001). Functional status of patients with oral cancer and its relation to style of coping, social support and psychological status. The British Journal of Oral & Maxillofacial Surgery, 39(5), 340–345. 10.1054/bjom.2001.0652 11601811

[23] Bao Y, Li L, Guan Y, Wang W, Liu Y, Wang P, et al (2016). Prevalence and associated positive psychological variables of anxiety and depression among patients with central nervous system tumors in China: a cross-sectional study. Psycho-Oncology. 10.1002/pon.4128 27072749

[24] Davis MP, Lagman R, Parala A, Patel C, Sanford T, Fielding F, et al (2017). Hope, symptoms, and palliative care. The American Journal of Hospice & Pa*lliative Care*, 34(3), 223–232. 10.1177/1049909115627772 26809826

[25] Rawdin B, Evans C, Rabow MW. (2013). The relationships among hope, pain, psychological distress, and spiritual well-being in oncology outpatients. Journal of Palliative Medicine, 16(2), 167–172. 10.1089/jpm.2012.0223 23101471 PMC3569921

[26] Xhulia D, Gerta J, Dajana Z, Koutelekos I, Vasilopoulou C, Skopelitou M, et al (2015). Needs of Hemodialysis Patients and Factors Affecting Them. *Global Journal of Health Science*, 8(6), 109–120. 10.5539/gjhs.v8n6p109 26755472 PMC4954872

[27] Luo Y-Y, Yu M-H, Hsieh L-Y. (2010). An exploration of education for patients with hemodialysis. *Leadership Nursing*, 11(1), 16–28.

[28] von Elm E, Altman DG, Egger M, Pocock SJ, Gotzsche PC, Vandenbroucke JP. (2014). The strengthening the reporting of observational studies in epidermiology (STROBE) statement: guidelines for reporting observational studies. *International Journal of Surgery*, 12, 1495–1499. 10.1016/j.ijsu.2014.07.013 25046131

[29] Weisbord SD, Fried LF, Arnold RM, Fine MJ, Levenson DJ, Peterson RA, Switzer GE. (2005). Prevalence, severity, and importance of physical and emotional symptoms in chronic hemodialysis patients. *Journal of The American Society of Nephrology*, 16(8), 2487–2494. 10.1681/ASN.2005020157 15975996

[30] Lee Y, Yang M-J., Lai T-J, Chiu N-M, Chau T-T. (2000). Development of the Taiwanese Depression Questionnaire. Chang Gung Medical Journal, 23(11), 688–694. 11190378

[31] Lin C-C, Liang C-C, Huang F-L, Lai Y-H. (2004). Relationships between hope and social support of hemodialysis patients. *Journal of Taiwan Nephrology Nursing Association*,3(1),17–31.

[32] Huang HM. (2003). *The investigation of stresses and demands for hemodialysis patients and family-taking one destrict education hospital in Chi-Yi example*. Nanhua University, Taiwan: Chi-Yi.

[33] Gorsuch RL. (1983). Factor analysis. Hillsdale, NJ: Lawrence Erlbaum.

[34] Chan M-F, Chan S-H, Day M-C. (2004). A pilot study on nurses' attitudes toward perinatal bereavement support: a cluster analysis. *Nurse Education Today*, 24(3), 202–210. 10.1016/j.nedt.2003.11.009 15046855 PMC7130498

[35] Dancey CP, Reidy J. (2007). *Statistics Without Maths for Psychology*. Pearson Education.

[36] Kao T-W, Tsai D-M, Wu K-D, Shiah C-J, Hsieh B-S, Chen W-Y.(2003). Impact of religious activity on depression and quality of life of chronic peritoneal dialysis patients in Taiwan. *Journal of The Formosan Medical Association*, 102(2), 127–130. 12709745

[37] Kao T-W, Lai M-S, Tsai T-J, Jan C-F, Chie W-C, Chen W-Y. (2009). Economic, social, and psychological factors associated with health-related quality of life of chronic hemodialysis patients in northern Taiwan: a multicenter study. *Artificial Organs*, 33(1), 61–68. 10.1111/j.1525-1594.2008.00675.x 19178442

[38] Cukor D, Cohen SD, Peterson RA, Limmel PL. (2007) Psychosocial aspects of chronic disease: ESRD as a paradigmatic illness. Journal of The American Society of Nephrology: JASN, 18(12), 3042–3055. 10.1681/ASN.2007030345 18003775

[39] Hawandeh S, Almari AM, Almutairi AS, Dator WLT. (2017). Determinants and prevalence of depression in patients with chronic renal disease, and their caregivers. *International Journal of Nephrology and Renovascular Disease*, 10, 183–189. 10.2147/IJNRD.S139652 28740416 PMC5503667

[40] Sugisawa H, Shimizu Y, Kumagai T, Sugisaki H, Ohira S, Shinoda T. (2016) Effects of socioeconomic status on physical and mental health of hemodialysis patients in Japan: differences by age, period, and cohort. *International Journal of Nephrology and Renovascular Diseas*e, 9:171–182. 10.2147/IJNRD.S107031 27471405 PMC4948840

[41] Gerogianni S, Babatsikou F, Gerogianni G, Koutis C, Panagiotou M, Psimenou E. (2016) Social life of patients undergoing haemodialysis. International Journal of Caring Sciences, 9 (1): 122–134.

[42] Chan MF, Wong FKY, Chow SKY. (2010). Investigating the health profile of patients with end-stage renal failure receiving peritoneal dialysis: a cluster analysis. *Journal of Clinical Nursing*, 19(5–6), 649–657. 10.1111/j.1365-2702.2009.03103.x 20500306

