# Supplementary figures and images for: Exploring demands of hemodialysis patients in Taiwan: A two-step cluster analysis
Source: PLoS One. 2020 Feb 7;15(2):e0228259. doi: 10.1371/journal.pone.0228259 (PMC7006915; doi:10.1371/journal.pone.0228259)

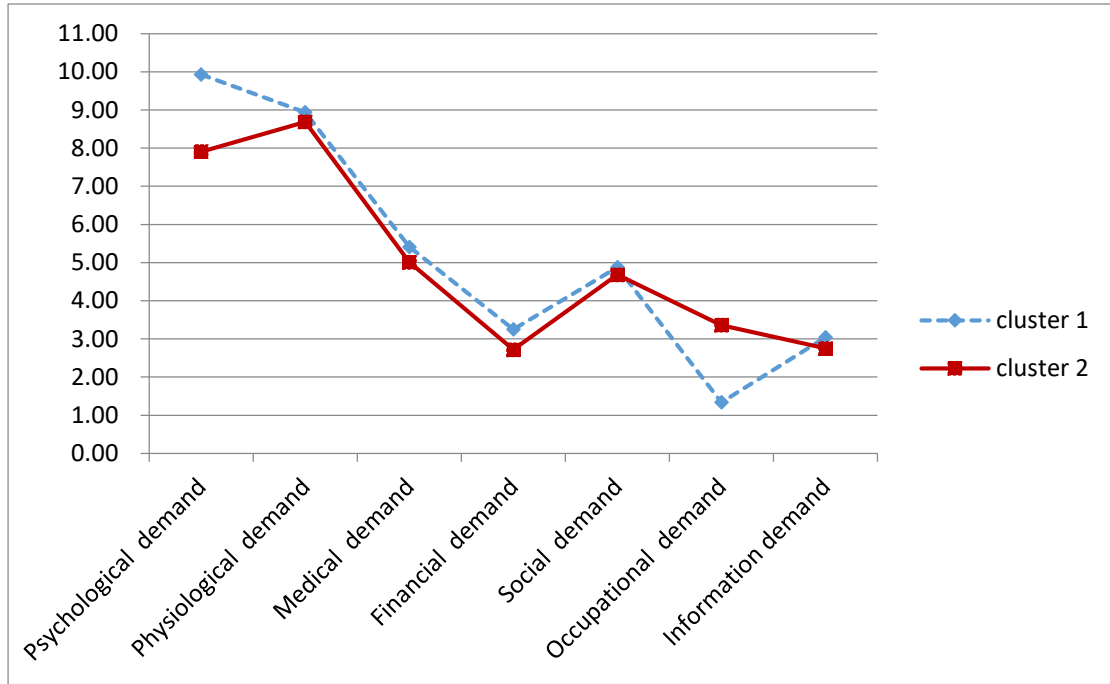

Supplement: S1 Fig — (PDF) [file pone.0228259.s001.pdf]
